# MAIT cell-directed therapy of *Mycobacterium tuberculosis* infection

**DOI:** 10.1038/s41385-020-0332-4

**Published:** 2020-08-18

**Authors:** Shunsuke Sakai, Keith D. Kauffman, Sangmi Oh, Christine E. Nelson, Clifton E. Barry, Daniel L. Barber

**Affiliations:** 1 T Lymphocyte Biology Section, Laboratory of Parasitic Diseases, National Institute of Allergy and Infectious Diseases, Bethesda, MD USA; 2 Tuberculosis Research Section, Laboratory of Clinical Immunology and Microbiology, National Institute of Allergy and Infectious Diseases, Bethesda, MD USA

## Abstract

Mucosal-associated invariant T (MAIT) cells are potential targets of vaccination and host-directed therapeutics for tuberculosis, but the role of MAIT cells during *Mycobacterium tuberculosis* (Mtb) infection in vivo is not well understood. Here we find that following Mtb infection MAIT cells mount minimal responses, and MAIT cell-deficient MR1^−/−^ mice display normal survival. Preinfection expansion of MAIT cells through 5-OP-RU vaccination fails to protect against subsequent Mtb challenge. In fact, 5-OP-RU vaccination delays Mtb-specific CD4 T cell priming in lung-draining lymph nodes, and conversely MR1 deficiency or blockade accelerates T cell priming. The MAIT cell-mediated delay in T cell priming is partly dependent on TGF-β. Surprisingly, 5-OP-RU treatment during chronic infection drives MAIT cell expansion and an IL-17A-dependent reduction in bacterial loads. Thus, during early infection MAIT cells directly contribute to the notoriously slow priming of CD4 T cells, but later during infection MAIT cell stimulation may be an effective host-directed therapy for tuberculosis.

## Introduction

Tuberculosis (TB) is the leading cause of death due to a single infectious agent^1^. The only vaccine currently available for TB, *Bacillus* Calmette-Guérin (BCG), provides little protection from TB beyond infancy as currently utilized, and new vaccination strategies are greatly needed. Antitubercular chemotherapy is effective in treating infection with drug-susceptible strains of *Mycobacterium tuberculosis* (Mtb), but new approaches in treating TB disease are required to meet the growing threat posed by drug resistant Mtb. Methodologies that manipulate host immune responses to treat TB, host-directed therapies (HDTs), hold great promise but none have been approved for clinical use^2^. A better understanding of host-protective immune cells and molecules may provide insight into targets for the development of novel vaccines and treatments for TB.

Several types of T cells restricted by class Ib molecules recognize mycobacterial antigens, and have a hypothetical advantage over conventional T cells as vaccine and therapy targets due to the relatively non-polymorphic nature of the restriction elements and abundance of these cells at mucosal surfaces^3^. Mucosal-associated invariant T (MAIT) cells are a particularly interesting potential target for TB vaccination and HDT. MAIT cells express a semi-invariant TCR specific for the riboflavin metabolite derivative 5-OP-RU presented by MR1. In support of their potential role in Mtb infection, MAIT cells are reduced in circulation and enriched in the airways of individuals with active TB disease compared to healthy donors^4–6^. MAIT cells represent the majority of human PBMCs that produce IFN-γ after in vitro restimulation with BCG, and BCG revaccination of Mtb-infected individuals after isoniazid preventative therapy boosts MAIT frequencies^7^. Intradermal BCG vaccination of macaques results in the upregulation of activation markers on MAIT cells^8^, and intravenous BCG vaccination induces pulmonary MAIT expansion in rhesus macaques^9^. In the mouse model, MAIT TCR transgenic mice displayed transiently reduced pulmonary Mtb loads during early infection^10^. Finally, it has been shown that MAIT cells can be dramatically expanded in vivo following the appropriate combination of antigenic and inflammatory stimuli^11^. However, the role of MAIT cells in host resistance to Mtb infection or their suitability as prophylactic or therapeutic targets in Mtb infection has not been formally established.

The MAIT TCR antigens presented by MR1 are not produced by the host, but rather by a fraction of the microbiota as well as several pathogenic fungi and bacteria including Mtb^12^. Under steady state conditions, MAIT cells likely receive persistent stimulation by microbiota-derived ligands. In fact, MAIT cells are dependent on commensal bacteria-derived riboflavin derivatives for selection in the thymus^13,14^, and MAIT cells have even been shown to regulate the composition of the intestinal microbiota^15^. Therefore, while MAIT cells are implicated in host resistance to infections, MAIT cells must distinguish between commensals and pathogens before deciding to exert inflammatory effector functions. Moreover, MAIT cells can have both pro-inflammatory as well as tissue repair functions after TCR stimulation and promote accelerated wound healing in the skin^14,16,17^. The inflammatory versus tissue repair functions exerted by MAIT cells are determined by the combination of TCR, cytokine, and costimulatory receptor signals received and are likely dynamically regulated during infection^18^. The current data on MAIT cell responses in vivo are in the context of acute, rapidly resolving infections^12^, and little is understood how MAIT cell function is regulated during the early and late stages of chronic infections such as Mtb. It is also not clear how this dual nature of MAIT cells impacts their utility as vaccine and therapeutic targets in different types of infections.

Here we find that the endogenous MAIT cell response has little role in host resistance to Mtb infection, and instead early MAIT cell responses impede the priming of conventional peptide-specific CD4 T cells. Moreover, stimulation of MAIT cells through administration of 5-OP-RU has dramatically different outcomes when done prior as compared to after infection. Preinfection MAIT cell vaccination does not protect against Mtb infection and leads to a further delay in CD4 T cell responses in a manner dependent on TGF-β. On the other hand, we also show that once Mtb infection is established, stimulating the MAIT TCR drives potent IL-17A-dependent reductions in bacterial loads. Thus, we show that MAIT cell responses are not a major part of normal host resistance to Mtb infection and that MAIT cell stimulation can lead to either TGF-β-dependent immunoregulation or IL-17A-dependent host protection depending on the timing relative to infection.

## Results

### Endogenous MAIT cells do not contribute to host protection following Mtb infection

**Fig. 1:**
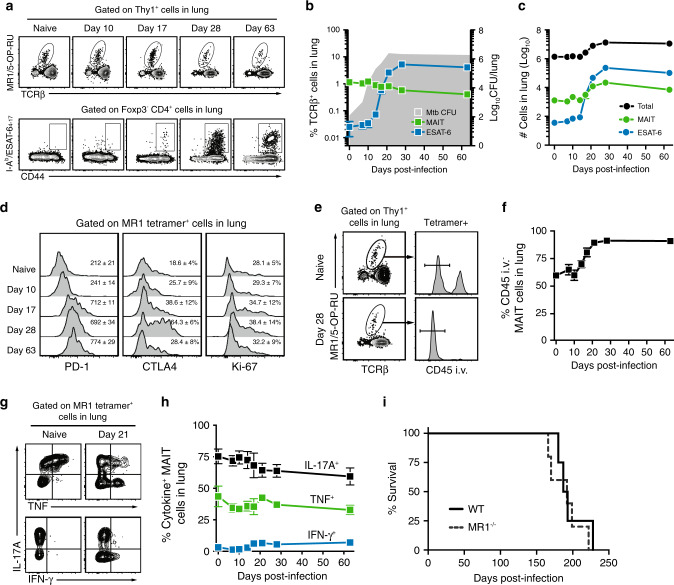
Endogenous MAIT cells do not contribute to host protection following Mtb infection. Wild-type (WT) C57BL/6 mice were infected with ~150 CFU of Mtb by aerosol and lungs were harvested at the indicated time points. **a** Representative FACS plots of MR1/5-OP-RU and I-A^b^/ESAT-6_4-17_ tetramer staining of lung cells. **b** Kinetics of the frequency of MAIT cells and I-A^b^/ESAT-6_4-17_^+^ CD4 T cells among TCRβ^+^ cells in the lungs. Bacterial CFU in the lungs are also shown. **c** Number of total lung cells, MAIT cells and I-A^b^/ESAT-6_4-17_^+^ CD4 T cells over time. **d** Representative histogram plots of PD-1, CTLA4, or Ki-67 expression by lung MAIT cells at the indicated time points. Numbers indicate the geometric MFI (PD-1) or % positive (CTLA4 and Ki-67) in the gated MAIT cells. **e** Representative FACS plots and histograms of CD45 intravascular (i.v.) staining of lung MAIT cells. **f** Kinetics of the frequency of CD45 i.v. staining negative (CD45 i.v.^−^) MAIT cells in the lungs. **g** Representative FACS plots of cytokine production by lung MAIT cells after stimulation with PMA and ionomycin. **h** Kinetics of the frequency of IL-17A-, TNF-, and IFN-γ-producing MAIT cells in the lungs. **i** Survival curve of WT and MR1^−/−^ mice (*n* = 5 per group) after aerosol Mtb infection. Date are representative of at least two experiments with three or more mice per time point or group. Error bars represent mean ± SEM.

To define the kinetics of MAIT cell responses in Mtb infection, mice were infected with Mtb and the frequency of MAIT cells in the lungs were assessed using MR1/5-OP-RU tetramers. In naive animals, MAIT cells comprise about 1–2% of lung TCRβ^+^ cells, which did not change after Mtb infection (Fig. 1a, b). In contrast, conventional I-A^b^/ESAT-6_4-17_-specific CD4 T cells were first detected in lungs ~17 days postinfection (p.i.) and outnumbered MAIT cells by 28 days p.i. (Fig. 1a, b). The number of MAIT cells in the lungs paralleled with the total number of lung cells and increased only after I-A^b^/ESAT-6_4-17_-specific CD4 T cells started to accumulate in lungs (Fig. 1c). MAIT cells showed an increased expression of PD-1 and CTLA4, but Ki-67 expression changed little after infection (Fig. 1d). We used an intravascular (i.v.) staining approach^19,20^ to distinguish cells in the tissue from those in the vasculature (Fig. 1e). In naive mice, ~70% of lung MAIT cells were negative for i.v. staining (i.v.^−^) and almost all MAIT cells became i.v.^−^ after 21 days (Fig. 1e, f), indicating that MAIT cells are most likely recruited into the lung after infection. Most of lung MAIT cells are IL-17A-producing MAIT17 cells which remained the dominant population throughout Mtb infection (Fig. 1g, h). Thus, MAIT cells upregulate activation markers and localize into the tissue but undergo minimal expansion and do not significantly change their effector capacity during Mtb infection.

Given the ability of MAIT cells to migrate into the lungs and produce IL-17A, we next asked whether MAIT cells contribute to host survival following Mtb infection. We found that MAIT cell-deficient MR1^−/−^ mice displayed similar survival times to WT mice after infection (Fig. 1i). Therefore, the endogenous MAIT cell response does not play a major role in host resistance against primary Mtb infection.

### MAIT cells delay the priming of Mtb-specific CD4 T cells in a MR1-dependent manner

**Fig. 2:**
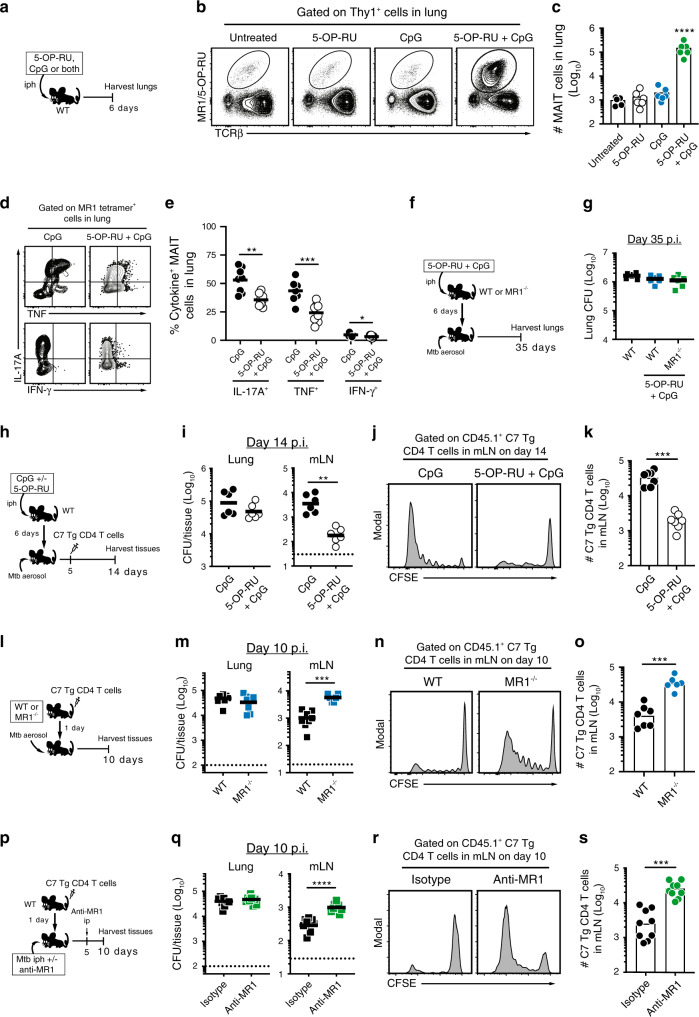
Prophylactic vaccination of MAIT cells delays the priming of Mtb-specific CD4 T cells in a MR1-dependent manner. **a** WT mice were treated intrapharyngeally (iph) with 1 μM 5-OP-RU, 10 μg of CpG, or both (in 30 μl of PBS per mouse). Lungs were harvested 6 days after treatment. Representative FACS plots of MR1/5-OP-RU tetramer staining of Thy1^+^ cells (**b**) and number of MAIT cells (**c**) in the lungs from mice treated as shown in **a**. Representative FACS plots (**d**) and frequency (**e**) of IL-17A^+^, TNF^+^, or IFN-γ^+^ lung MAIT cells after treatment with CpG ± 5-OP-RU. **f** WT mice were iph treated with CpG ± 5-OP-RU 6 days prior to aerosol Mtb infection and lungs were harvested on day 35 postinfection (p.i.). **g** Bacterial CFU in the lungs from mice treated as shown in **f**. **h** WT mice were iph treated with CpG ± 5-OP-RU 6 days prior to aerosol Mtb infection and received intravenously CFSE-labeled C7 transgenic (Tg) CD4 T cells on day 5 p.i. Lungs and mediastinal LN (mLN) were harvested on day 14 p.i. Bacterial CFU in the tissues (**i**), representative histograms of CFSE dilution (**j**), and number (**k**) of C7 CD4 T cells in the mLN from mice treated as shown in **h**. **l** CFSE-labeled C7 CD4 T cells were adoptively transferred into WT or MR1^−/−^ mice 1 day before aerosol Mtb infection and tissues were harvested on day 10 p.i. **m**–**o** Bacterial CFU in the tissues (**m**), representative histograms of CFSE dilution (**j**), and number (**k**) of C7 CD4 T cells in the mLN from mice treated as shown in **l**. **p** CFSE-labeled C7 CD4 T cells were adoptively transferred into WT mice prior to iph infection with Mtb. Mice were treated with isotype or anti-MR1 antibody at the time of infection and 5 days p.i. Tissues were harvested on day 10 p.i. Bacterial CFU in the tissues (**q**), representative histograms of CFSE dilution (**r**), and number (**s**) of C7 CD4 T cells in the mLN from mice treated as shown in **p**. Date are pooled from two independent experiments with three or more mice per group. *p < 0.05, **p < 0.01, ***p < 0.001, ****p < 0.0001.

Although MAIT cells did not affect the outcome of infection in this model, we next asked if targeting MAIT cells with a pre-exposure vaccine could be effective against Mtb infection. Naive mice were treated with an intrapharyngeal (iph) inoculation of 5-OP-RU, CpG alone or both, and MAIT cell expansion in the lungs was measured after 6 days (Fig. 2a). Consistent with the previous finding^11^, treatment with either 5-OP-RU or CpG alone did not expand MAIT cells, but 5-OP-RU+CpG vaccination resulted in as much as a 100-fold expansion of MAIT cells in the lungs (Fig. 2b, c). MAIT17 cells remained the dominant subset in lungs after vaccination (Fig. 2d, e). To test if prophylactic vaccination of MAIT cells confers protection against Mtb infection, WT or MR1^−/−^ mice were vaccinated with 5-OP-RU+CpG and then challenged with Mtb (Fig. 2f). On day 35 p.i. there was no difference in bacterial loads in the lungs between untreated and MAIT cell-vaccinated WT mice (Fig. 2g). Furthermore, vaccinated MR1^−/−^ mice had similar bacterial loads in the lungs compared to WT animals (Fig. 2g). Taken together, these data indicate that prophylactic vaccination of MAIT cells does not provide protection against Mtb infection in mice.

Given that MAIT cells are pre-positioned in the lungs prior to infection, it has been speculated that MAIT cells may respond very early to Mtb infection and that targeting MAIT cells could be used as an adjuvant to enhance conventional T cell responses^3^. Therefore, we next examined the contribution of MAIT cells to the early control of Mtb infection as well as their impact on the priming of conventional peptide-specific CD4 T cells. As shown in Fig. 2h, mice were treated with CpG alone or 5-OP-RU+CpG 6 days prior to aerosol Mtb infection and CFSE-labeled I-A^b^/ESAT-6 specific TCR transgenic (C7) CD4 T cells were adoptively transferred into the infected mice 5 days later to track T cell priming. On day 14 p.i. lungs and mediastinal lymph nodes (mLNs) were isolated from the mice. Interestingly, although MAIT cell vaccination leads to a robust expansion of MAIT cells in lungs at the time of infection (Fig. 2c), we did not observe any protective effect of the vaccination for controlling early Mtb growth in the lungs (Fig. 2i). Instead, the bacterial loads in mLNs of vaccinated mice were significantly reduced compared to control mice (Fig. 2i). Bacterial dissemination from the lung to draining LNs is critical for the initiation of CD4 T cell responses^21–23^, so we next analyzed the impact of MAIT cell vaccination on the priming of Mtb-specific CD4 T cells in the mLN. In control mice, C7 cells expanded extensively by day 14 in the mLNs, while C7 cells in the vaccinated mice displayed little proliferation at this time point (Fig. 2j, k). Therefore, not only does prophylactic vaccination of MAIT cells fail to enhance control of Mtb infection, it also delays bacterial dissemination to the draining LNs and subsequent CD4 T cell priming.

We next asked if MAIT cell deficiency modulates early bacterial dissemination to lung-draining LNs and CD4 T cell priming after Mtb infection. CFSE-labeled C7 CD4 T cells were adoptively transferred into WT and MAIT cell-deficient MR1^−/−^ mice, and 10 days following Mtb infection the tissues were collected (Fig. 2l). There was no difference in bacterial numbers in lungs of WT and MR1^−/−^ mice, but MR1^−/−^ mice showed higher CFU in the mLNs (Fig. 2m). On day 10 p.i. the donor C7 cells had not yet expand in WT mice, while in MR1^−/−^ recipients the C7 cells had already proliferated extensively (Fig. 2n, o). MAIT cells can be activated via either TCR or cytokine-dependent mechanisms^12^, so we next asked whether MAIT cells require MR1-dependent TCR stimulation to delay CD4 T cell priming after Mtb infection. To this end, C7 CD4 T cells were transferred into WT mice, then infected recipients were treated with anti-MR1 blocking antibody, and the tissues were analyzed on day 10 p.i. (Fig. 2p). Similar to the results observed in MR1^−/−^ mice (Fig. 2m), MR1 blockade had no effect on pulmonary bacterial loads, but increased CFU numbers in the mLNs compared to control mice (Fig. 2q). Furthermore, MR1 blockade led to accelerated priming of C7 CD4 T cells in the mLNs at day 10 (Figs. 2r, s). Collectively, these data show that MAIT cell deficiency accelerates, while increased MAIT cell numbers further delays conventional CD4 T cell priming. Therefore, MAIT cells, driven by TCR stimulation, directly play a role in the slow induction of adaptive immune responses after Mtb infection.

### MAIT cell-mediated delay in CD4 T cell priming is partially reversed by TGF-β blockade

**Fig. 3:**
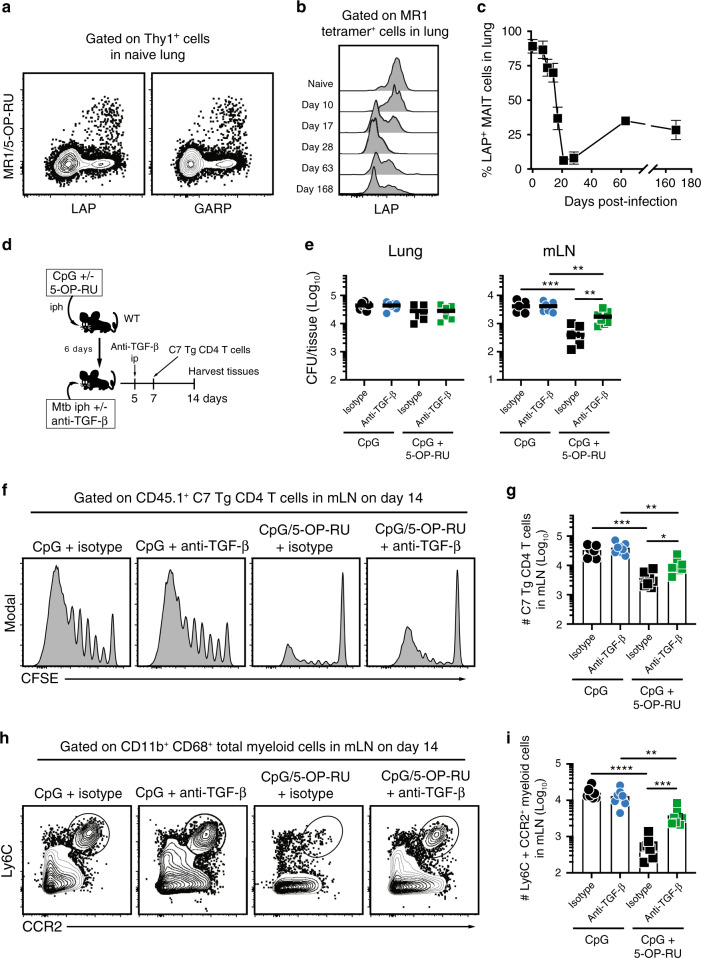
MAIT cell-dependent delay in CD4 T cell priming is partially reversed by TGF-β blockade. **a** Representative FACS plots of LAP and GARP expression on lung MAIT cells in naive mouse. **b** Representative histograms of LAP expression on lung MAIT cells at the indicated time points. **c** Frequency of LAP^+^ MAIT cells in the lungs over time. **d** WT mice were iph treated with CpG ± 5-OP-RU 6 days before iph Mtb infection. Mice were treated with isotype or anti-TGF-β antibody at the time of infection and 5 days p.i. CFSE-labeled C7 CD4 T cells were adoptively transferred into mice on day 7 p.i. and tissues were harvested on day 14 p.i. Bacterial CFU in the tissues (**e**), representative histograms of CFSE dilution (**f**), and number (**g**) of C7 CD4 T cells in the mLN from mice treated as shown in **d**. Representative FACS plots (**h**) and frequency (**i**) of CCR2^+^Ly6C^+^ myeloid cells in the mLN from mice treated as shown in **d**. Date are pooled from two independent experiments with three or four mice per group. Error bars represent mean ± SEM. *p < 0.05, **p < 0.01, ***p < 0.001, ****p < 0.0001. See also Fig. S1.

Upon TCR stimulation MAIT cells express genes related to tissue repair^14,16,17^, so we next asked if an immunoregulatory function of MAIT cells results in delayed adaptive immunity after Mtb infection. We found that MAIT cells express high levels of cell surface TGF-β, measured in the form of latency-associated peptide (LAP), as well as GARP, the molecule that tethers TGF-β to the cell surface (Fig. 3a). In fact, lung parenchymal MAIT cells expressed the highest levels of these molecules compared to other lung lymphocyte subsets (Fig. S1A–C). LAP expression decreased as the infection progressed (Fig. 3b, c). To test the role of TGF-β in the delayed CD4 T cell priming induced by MAIT cells, vaccinated mice were treated with anti-TGF-β neutralizing antibodies starting at the time of infection and bacterial growth and the proliferation of CFSE-labeled C7 T cells was measured 14 days p.i. (Fig. 3d). TGF-β blockade did not affect Mtb growth in the lungs but increased bacterial loads in the mLN of mice vaccinated with CpG+5-OP-RU (Fig. 3e). Indeed, this increased Mtb dissemination to the mLN after TGF-β blockade was accompanied by a partial recovery of C7 CD4 T cell expansion (Fig. 3f, g). Importantly, TGF-β blockade did not accelerate bacterial dissemination into mLN and C7 T cell priming in MR1^−/−^ mice (Fig. S1D–G), suggesting that TGF-β is required for MAIT cell-dependent delay of CD4 T cell priming after Mtb infection.

CCR2^+^Ly6C^+^ monocytes are required for the trafficking of Mtb bacilli from the lungs to mLN and the subsequent the priming of Mtb-specific CD4 T cells^24^. Therefore, we examined the role of MAIT cells and TGF-β on the migration of CCR2^+^Ly6C^+^ myeloid cells into the mLN. In unvaccinated control mice, TGF-β blockade had no impact on the numbers of CCR2^+^Ly6C^+^ myeloid cells in the mLNs on day 14 p.i. (Fig. 3h, i). Strikingly, MAIT cell vaccination with CpG+5-OP-RU dramatically reduced the number of CCR2^+^Ly6C^+^ myeloid cells in the mLN (Fig. 3h, i). Moreover, TGF-β blockade partially restored the number of CCR2^+^Ly6C monocytes in the draining LNs of MAIT cell-vaccinated mice (Fig. 3h, i). Taken together, prophylactic vaccination of MAIT cells leads to TGF-β-dependent suppression of CCR2^+^Ly6C^+^ myeloid cell trafficking into the mLNs early after Mtb infection which likely results in the delayed priming of Mtb-specific CD4 T cells. We should point out, however, that we have not directly measured the trafficking of bacteria via these monocytes and cannot rule out the possibility that another cell type was responsible for the increased dissemination of bacteria to the draining nodes.

### Therapeutic vaccination of MAIT cells enhances control of chronic Mtb infection

**Fig. 4:**
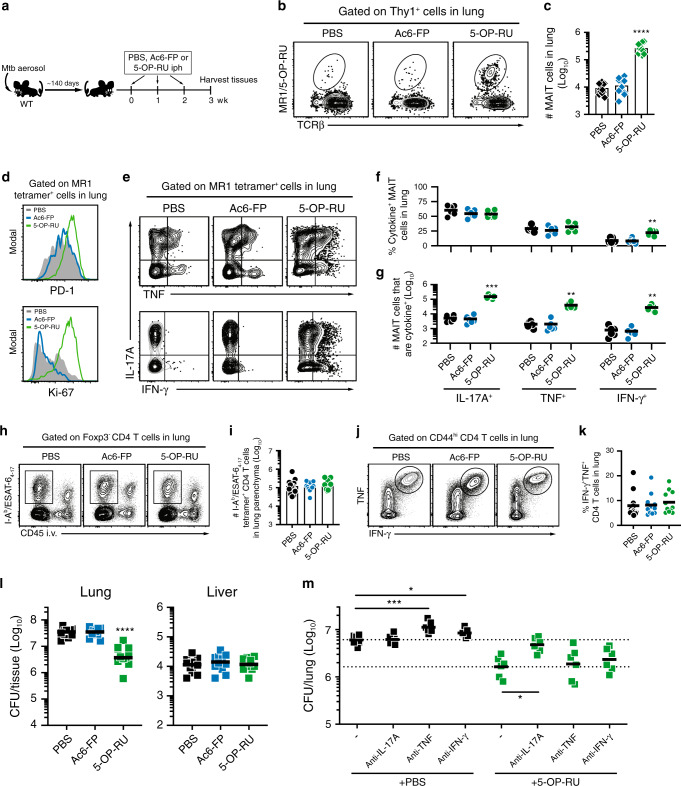
Therapeutic vaccination of MAIT cells during Mtb infection. **a** Mtb-infected WT mice (~140 days p.i.) were iph treated with PBS, Ac6-FP, or 5-OP-RU once a week for 3 weeks. Tissues were harvested a week after the last treatment. Representative FACS plots (**b**) and number (**c**) of MAIT cells in the lungs. **d** Representative histogram plots of PD-1 or Ki-67 expression by lung MAIT cells. Representative FACS plots (**e**), frequency (**f**), and number (**g**) of IL-17A^+^, TNF^+^, or IFN-γ^+^ MAIT cells in the lungs. Representative FACS plots (**h**) and number (**i**) of I-A^b^/ESAT-6_4-17_ tetramer^+^ CD4 T cells in the lungs. Representative FACS plots (**j**) and number (**k**) of TNF^+^IFN-γ^+^ lung CD4 T cells after stimulation with ESAT-6_1-20_ peptides. **l** Bacterial CFU in the tissues 3 weeks after treatment with PBS, Ac6-FP, or 5-OP-RU. **m** Lung CFU 3 weeks after treatment with PBS or 5-OP-RU along with the indicated neutralizing antibodies. Data are pooled from at least two independent experiments with three or four mice per group. *p < 0.05, **p < 0.01, ***p < 0.001, ****p < 0.0001. See also Fig. S2.

Lastly, we examined the impact of MAIT cell stimulation during chronic Mtb infection. WT mice were infected with Mtb and starting ~140 days p.i. the mice were iph treated with PBS, acetyl-6-formylpterin (Ac6-FP, an inhibitory ligand for MAIT cells) or 5-OP-RU once a week for 3 weeks (Fig. 4a). We found that pulmonary instillation of Ac6-FP had no effect on the MAIT cell numbers in the lungs (Fig. 4b–f). By contrast, 5-OP-RU administration led to an ~30-fold increase in the MAIT cell numbers (Fig. 4c) and upregulation of PD-1 and Ki-67 compared to control mice (Fig. 4d). Furthermore, 5-OP-RU treatment increased the numbers of both MAIT17 and MAIT1 cells in the lungs by approximately tenfold (Fig. 4e–g). Of note, neither Ac6-FP nor 5-OP-RU treatment impacted conventional Mtb-specific CD4 T cell number or function in the lungs (Fig. 4h–k). Strikingly, however, we found that 3 weeks of 5-OP-RU treatment resulted in an ~1 log reduction in bacterial loads in the lung but not liver (Fig. 4l). Importantly, the therapeutic effect of 5-OP-RU instillation during Mtb infection was MR1 dependent, as 5-OP-RU treatment did not enhance bacterial clearance in MR1^−/−^ mice (Fig. S2).

To examine the mechanism of this MAIT-directed therapy of Mtb infection, mice were treated with either anti-IL-17A, anti-TNF, or anti-IFN-γ neutralizing antibodies along with 5-OP-RU instillation. As expected, TNF or IFN-γ blockade during chronic Mtb infection led to an increase in the lung bacterial loads in PBS-treated mice, but neither TNF nor IFN-γ blockade prevented the enhanced bacterial clearance after 5-OP-RU treatment (Fig. 4m). In contrast, IL-17A blockade had no effect in control mice but significantly increased Mtb loads in the lungs of 5-OP-RU-treated mice (Fig. 4m). Thus, the repeated stimulation of MAIT cells during chronic Mtb infection drives robust expansion of MAIT cells and enhances IL-17A-dependent bacterial clearance in the lung.

## Discussion

MAIT cells have been shown to protect against microbial infections including *Escherichia* *coli*, *Mycobacterium* *abscessus*, *Francisella* *tularensis*, *Legionella* *longbeachae,* and influenza virus infection^25–28^. Despite previous evidence showing that MAIT cells can respond to Mtb infection, here we find that in mice the endogenous MAIT cell response in the lungs is relatively weak and has no discernible protective role. This is consistent with reports that pulmonary MAIT cells display few markers of activation and do not accumulate in the granulomas of Mtb-infected rhesus macaques^29,30^. However, here we also show that stimulating MAIT cells through their TCR by exogenous administration of 5-OP-RU during chronic infection results in a surprising reduction in bacterial loads. In fact, MAIT stimulation with 5-OP-RU is a far superior to therapeutic vaccination with BCG, which is detrimental^31^, or injection of CD4 T cell antigenic peptides, which has minimal protective efficacy^32,33^. Overall, these data show MAIT cells can display potent antitubercular activity in vivo but only if TCR ligand availability is increased through exogenous administration.

Although there may be great utility in a postexposure TB vaccine, prophylactic vaccination stands to make the greatest impact on the global TB burden. Unfortunately, we found that preexposure MAIT cell vaccination failed to enhance control of Mtb challenge in this murine model. Moreover, we found that MAIT cells have an unexpected role in inhibiting the induction of conventional T cell responses to Mtb infection. The priming of Mtb-specific T cell responses after aerosol exposure is slow compared to other pathogens^34^. The mechanisms underlying the lag in T cell priming are poorly understood but are thought to result from slow bacterial replication and the time it takes to deliver bacteria to the lung-draining LN. Indeed, T cell responses to Mtb infection are not initiated until bacilli are trafficked to the pulmonary LNs by CCR2^+^ monocytes^24,35,36^ and accelerated delivery of bacteria to the mLNs leads to earlier T cell priming^37^. Here we provide evidence that the kinetic of this process is regulated by MAIT cells. Our data indicate that, during the first weeks of Mtb infection MAIT cells may suppress CCR2^+^ myeloid cell migration and bacterial dissemination to the mLNs along with the subsequent priming of Mtb-specific CD4 T cell responses. It is important to point out that the changes in the rate of T cell priming were not sufficient to alter the outcome of the infection in this setting, as neither the accelerated T cell responses in the absence of MAIT cells nor the extended delay of pulmonary T cell responses after boosting MAIT cells impacted lung bacterial loads relative to controls. These results also contrast with *F. tularensis* infection, where MAIT cells have been shown to play an important role in promoting CD4 T cell responses in part through their promotion of CCR2^+^ monocyte differentiation into dendritic cells^38^. The mechanisms underlying these seemingly opposing roles of early MAIT cell responses in different pulmonary infections are not clear but may be linked to the presumed differences in 5-OP-RU production between Mtb and *F. tularensis*.

The mechanisms of MAIT cell-dependent modulation of conventional peptide-specific CD4 T cell responses may reflect their role in tissue homeostasis. We showed that MAIT cells express high levels of LAP and GARP, and the delayed T cell priming in the lung-draining LNs can be partly reversed by TGF-β blockade. However, many lymphocyte subsets in the lungs express cell surface TGF-β, and we have not shown that MAIT cell-derived TGF-β directly contributes to this effect. Several recent papers have shown that MAIT cells can display tissue repair/immunoregulatory phenotypes, including TGF-β expression, alongside their pro-inflammatory functions^14,16,17^, and it has also been shown that MAIT cells can suppress EAE in an IL-10-dependent manner^39^. Thus, our results support the emerging concept that MAIT cells have immunoregulatory roles in some settings, but further studies are needed to better define the inhibitory functions of MAIT cells in Mtb infection.

It is not clear why MAIT cell stimulation was only beneficial during but not prior to Mtb infection, but it may relate to their activation status. It is possible that at homeostasis the tissue repair/immunoregulatory features of MAIT cells are dominant, while during infection, innate inflammatory signals license MAIT cells to switch into a potent antimicrobial effector state. Here, we used CpG+5-OP-RU to trigger MAIT cell expansion in naive mice, but during chronic Mtb infection 5-OP-RU alone was sufficient to drastically expand MAIT cells. Moreover, we showed that TGF-β expression is very high on MAIT cells in naive mice and was rapidly lost after Mtb infection. Thus, we speculate that the low dose of infection, slow bacterial growth, and lack of overt inflammatory response during the first weeks of Mtb infection allows the homeostatic functions of MAIT cells to predominate leading to suppression of conventional T cell priming. In the later stages of Mtb infection, the inflammatory environment may provide the additional signals required for MAIT cells to expand and mediate antimicrobial effector functions upon TCR triggering. This hypothesis, however, remains to be formally tested.

The enhanced bacterial control after 5-OPR-U was primarily IL-17A dependent. Although MAIT cells are a major source of IL-17A in mice, and we have treated the animals with a MAIT TCR ligand, we should point out that we have not formally shown MAIT cells are the source of the host protective IL-17A after 5-OP-RU administration. The cellular targets and downstream mediators of IL-17A-dependent control of Mtb infection after 5-OP-RU administration are also not clear. We started the 5-OP-RU treatment during a phase when bacterial loads are relatively stable, so the reduction in CFU likely resulted from IL-17-mediated bactericidal activity. We should also point out that IL-17A blockade only partly abrogated the protective effect, so it is possible that MAIT-dependent control is due to the function of multiple effector pathways.

Although there is overlap between the gene expression pattern between murine and human MAIT cells, there are also major differences^12^. For example, murine MAIT cells are primarily IL-17A-producing CD4^−^CD8^−^ cells, while the majority of human and macaque MAIT cells are CD8^+^ and secrete IFN-γ. Therefore, it will be critical to the validate these results using larger animal models such as guinea pigs, rabbits, or macaques. This is especially true given the primary role of IL-17A in the protective effect in mice, and its relatively lower expression by human MAIT cells. It will also be important to develop delivery strategies whereby MAIT cells could be most effectively and selectively boosted for the treatment of TB (e.g., inhalation of stabilized MAIT ligands). Nonetheless, these data indicate that targeting MAIT cells may hold promise as a therapeutic vaccine/HDT for TB.

## Materials and methods

### Mice

Six-to-twelve-week-old male and female B6 (C57BL/6NTac; CD45.2^+^, Thy1.2^+^), CD45.1 (B6.SJL; CD45.1^+^, Thy1.2^+^), Thy1.1 (B6.PL-Thy1a/CyJ; CD45.2^+^, Thy1.1^+^) mice were obtained through a supply contract between the NIAID/NIH and Taconic Farms. MR1^−/−^ mice originally generated by Dr. Susan Gilfillan (Washington University, St Louis School of Medicine, MO) were kindly provided from Dr. Yasmine Belkaid (NIAID/NIH, MD). C7 TCR transgenic mice, expressing a TCR specific for ESAT-6_3-17_, were generously donated by Dr. Eric Pamer (Memorial Sloan Kettering Cancer Center, NY) and were bred on the CD45.1^+^ congenic B6 backgrounds. All animals were housed at the AAALAC International-accredited BSL3 facility at the NIAID in accordance with the National Research Council Guide for the Care and Use of Laboratory Animals. All technical procedures and experimental endpoints were approved by the NIAID Division of Intramural Research Animal Care and Use Committee and listed in the animal study proposal LPD-24E.

### Aerosol infections

Mice were exposed to ~150 CFU of Mtb H37Rv strain using an aerosol inhalation exposure system (Glas-Col, LLC). Infection doses were measured by serial dilutions of lung homogenates on 7H11 agar plates supplemented with oleic acid-albumin-dextrose-catalase (Difco) immediately post exposure.

### Synthetic procedure for 5-OP-RU

The synthesis of 5-OP-RU was started from commercially available d-(-)-ribose and 6-chloro-5-nitropyrimidine-2,4(1*H*,3*H*)-dione following the previously reported procedure with some modification^40–42^. After the formation of 5-nitro-6-(((2*S*,3*S*,4*R*)-2,3,4,5-tetrahydroxypentyl)amino)pyrimidine-2,4(1*H*,3*H*)-dione, to a suspension of this compound (0.1 g, 0.33 mmol) in 3 ml of H_2_O was added 10 mg of 10% palladium on carbon (10% w/w). The reaction mixture was stirred overnight under H_2_ atmosphere at room temperature. After filtration of the reaction mixture through a pad of celite, it was freeze dried to obtain 5-A-RU as a white solid. Without further purification, 5-A-RU was directly used for the next reaction. To a solution of 5-A-RU in 5 ml of anhydrous DMSO was added methylglyoxal (40% in H_2_O, 0.36 mmol). The reaction mixture was stirred overnight at room temperature. After lyophilization, the crude mixture was immediately purified using Varian preparative HPLC system equipped with a reverse phase column (Phenomenex Luna C18, 10 μm, 250 × 21.2 mm) in the condition of 20 ml/min of flow rate with linear gradient over 30 min (solvent A was Milli-Q water and B was 80% aqueous acetonitrile, the detection wavelength was 365 nm). Due to the stability issue, both eluents did not contain any salts. After the collection of the product peaks, the solution was immediately frozen and lyophilized in a clean environment to obtain the desired 5-OP-RU as a solid (12.9 mg, 12%). For the biological test, 5-OP-RU was dissolved in DMSO to make 10 mM stock solution. All reagents including solvents used in the synthetic procedure were purchased from Sigma-Aldrich.

### Intrapharyngeal treatment

For therapeutic vaccination, infected mice were anesthetized with isoflurane, suspended vertically, and their tongues were pulled aside to expose the pharynx^43^, and then 1 μM 5-OP-RU or Ac6-FP (Cayman Chemical) diluted in 30 μl of PBS was administrated iph into the airway once in a week for 3 weeks. Alternatively, mice were treated with combination of 200 μg each of neutralizing antibody for cytokine blockade. In prophylactic vaccination experiments, iph inoculation with 5-OP-RU or CpG (10 μg of ODN1826, InvivoGen) alone or both in 30 μl of PBS was performed on naive mice. In some experiments, mice were inoculated iph with ~150 CFU of Mtb in combination with 20 μg of isotype, anti-MR1 (26.5, BioLegend), or anti-TGF-β antibody (1D11.16.8, Bio X cell) in 30 μl of PBS on day 0, and 100 μg each of antibody was given via intraperitoneally on day 5 p.i.

### C7 CD4 T cell adoptive transfers

Spleens and LNs were harvested from naive C7 TCR transgenic mice and mashed through 70-μm cell strainer. After ACK red blood cell lysis, CD4 T cells were positively selected using MACS magnetic beads and columns (Miltenyi Biotec). Purified cells were labeled with 1 μM CFSE (Thermo Fisher) at 37 °C for 10 min and washed. Two million C7 CD4 T cells were transferred intravenously into each recipient.

### Intravascular staining and tissue processing

Labeling cells in vasculature was performed as previously described^19,20^. Mice were injected with 2.5 μg of anti-CD45 fluorochrome-labeled antibody (30-F11), and after 3 min, animals were euthanized by cervical dislocation. Lungs were harvested and minced using a gentleMACs dissociator (Miltenyi Biotec) and were enzymatically digested in a shaker incubator at 37 °C for 40 min in RPMI medium containing 1 mg/ml Collagenase D (Roche-Diagnostics), 1 mg/ml hyaluronidase, 50 U/ml DNase I, and 1 mM aminoguanidine (all from Sigma-Aldrich). Suspensions were then passed through a 100-μm cell strainer and enriched for lymphocytes using a 37% Percoll density gradient centrifugation. Mediastinal LN cell suspensions were prepared using gentle disruption of the organs through a 70-μm cell strainer.

### In vitro stimulation

Cells were stimulated in complete medium containing 10% FCS at 1 × 10^7^ cells/ml at 37 °C with either PMA and ionomycin (Leukocyte activation cocktail, BD Biosciences) for 3 h or ESAT-6_1-20_ peptide for 5 h in the presence of brefeldin A, monensin (Thermo Fisher), and 1 mM aminoguanidine. Cells were assessed for intracellular cytokine production as described below.

### Flow cytometry

Tetramer stains were performed by incubating 1 × 10^6^ cells at 37 °C for 30 min with either MR1/5-OP-RU or I-A^b^/ESAT-6_4-17_ tetramer in complete medium containing 10% FCS, monensin, and 1 mM aminoguanidine. CD1d/PBS-57 tetramer was stained at 4 °C. Tetramers were produced by the NIAID tetramer core facility (Emory University, GA). Cells were stained with fluorochrome-labeled antibodies against Thy1.1 (OX-7), Thy1.2 (30H-12), CD45.1 (A20), CD45.2 (104), TCRβ (H57-597), TCRγ/δ (GL3), CD3e (145-2C11), CD4 (RM4-4), CD8 (53-6.7), CD44 (IM7), KLRG1 (2F1), PD-1 (29 F.1A12), LAP (TW7-16B4), GARP (F011-5), Nk1.1 (PK136), Ly6G (1A8), Ly6C (HK1.4), CCR2 (890231), CD11b (M1/70), and Fixable Viability Dye eFluor 780 for 20 min at 4 °C and washed. Cells were then fixed and permeabilized using the Foxp3/Transcriptional Factor staining kit and stained with fluorochrome-labeled antibodies against Foxp3 (FJK-16s), Ki-67 (B56), CTLA4 (UC10-4B9), CD68 (FA-11), IL-17A (TC11-18H10.1), TNF (MP6-XT22), and IFN-γ (XMG1.2) for a hour at 4 °C. All antibodies were purchased from BioLegend, Thermo Fisher, BD Biosciences, and R&D Systems. Data for all samples were collected on a BD FACSymphony A5 and analyzed using FlowJo version 10 software.

## Quantification and statistical analysis

All analyses were conducted using GraphPad Prism version 8 software. Mice group sizes were not determined by statistical tests and were based on the number of animals that can be housed per cage. Mice were assigned to experimental groups as available and not were randomized. The study was not performed blinded. Two-sample *t* test was used for two group comparisons and ANOVA was used for comparing multiple groups. The tests were two-tailed and all our data met the assumptions of the test. Unless specifically denoted in the Figures, a *p* value < 0.05 was considered statistically significant. Data are presented as mean ± SEM.

## Supplementary information

Supplementary Figures

## References

[1] WHO. Global tuberculosis report 2019. https://www.who.int/tb/publications/global_report/en/ (2019).

[2] Wallis RS, Hafner R (2015). Advancing host-directed therapy for tuberculosis. Nat. Rev. Immunol..

[3] Joosten SA (2019). Harnessing donor unrestricted T-cells for new vaccines against tuberculosis. Vaccine.

[4] Vorkas, C. K. et al. Mucosal-associated invariant and gammadelta T cell subsets respond to initial *Mycobacterium tuberculosis* infection. *JCI Insight* **3**, e121899 (2018). 10.1172/jci.insight.121899 PMC6237486 30282828

[5] Kwon YS (2015). Mucosal-associated invariant T cells are numerically and functionally deficient in patients with mycobacterial infection and reflect disease activity. Tuberculosis.

[6] Wong EB (2019). TRAV1-2(+) CD8(+) T-cells including oligoconal expansions of MAIT cells are enriched in the airways in human tuberculosis. Commun. Biol..

[7] Suliman S (2019). MR1-independent activation of human mucosal-associated invariant T cells by mycobacteria. J. Immunol..

[8] Greene JM (2017). MR1-restricted mucosal-associated invariant T (MAIT) cells respond to mycobacterial vaccination and infection in nonhuman primates. Mucosal Immunol..

[9] Darrah PA (2020). Prevention of tuberculosis in macaques after intravenous BCG immunization. Nature.

[10] Sakala IG (2015). Functional heterogeneity and antimycobacterial effects of mouse mucosal-associated invariant T cells specific for riboflavin metabolites. J. Immunol..

[11] Chen Z (2017). Mucosal-associated invariant T-cell activation and accumulation after in vivo infection depends on microbial riboflavin synthesis and co-stimulatory signals. Mucosal Immunol..

[12] Godfrey DI, Koay HF, McCluskey J, Gherardin NA (2019). The biology and functional importance of MAIT cells. Nat. Immunol..

[13] Legoux F (2019). Microbial metabolites control the thymic development of mucosal-associated invariant T cells. Science.

[14] Constantinides MG (2019). MAIT cells are imprinted by the microbiota in early life and promote tissue repair. Science.

[15] Smith AD (2019). Microbiota of MR1 deficient mice confer resistance against *Clostridium difficile* infection. PLoS One.

[16] Leng T (2019). TCR and inflammatory signals tune human MAIT cells to exert specific tissue repair and effector functions. Cell Rep..

[17] Hinks TSC (2019). Activation and in vivo evolution of the MAIT cell transcriptome in mice and humans reveals tissue repair functionality. Cell Rep..

[18] Berkson JD, Prlic M (2017). The MAIT conundrum—how human MAIT cells distinguish bacterial colonization from infection in mucosal barrier tissues. Immunol. Lett..

[19] Anderson KG (2014). Intravascular staining for discrimination of vascular and tissue leukocytes. Nat. Protoc..

[20] Galkina E (2005). Preferential migration of effector CD8+ T cells into the interstitium of the normal lung. J. Clin. Investig..

[21] Wolf AJ (2008). Initiation of the adaptive immune response to *Mycobacterium tuberculosis* depends on antigen production in the local lymph node, not the lungs. J. Exp. Med..

[22] Reiley WW (2008). ESAT-6-specific CD4 T cell responses to aerosol *Mycobacterium tuberculosis* infection are initiated in the mediastinal lymph nodes. Proc. Natl Acad. Sci. USA.

[23] Gallegos AM, Pamer EG, Glickman MS (2008). Delayed protection by ESAT-6-specific effector CD4+ T cells after airborne *M. tuberculosis* infection. J. Exp. Med..

[24] Samstein M (2013). Essential yet limited role for CCR2(+) inflammatory monocytes during *Mycobacterium tuberculosis*-specific T cell priming. Elife.

[25] Le Bourhis L (2010). Antimicrobial activity of mucosal-associated invariant T cells. Nat. Immunol..

[26] Meierovics A, Yankelevich WJ, Cowley SC (2013). MAIT cells are critical for optimal mucosal immune responses during in vivo pulmonary bacterial infection. Proc. Natl Acad. Sci. USA.

[27] Wang H (2018). MAIT cells protect against pulmonary *Legionella longbeachae* infection. Nat. Commun..

[28] van Wilgenburg B (2018). MAIT cells contribute to protection against lethal influenza infection in vivo. Nat. Commun..

[29] Bucsan AN (2019). Mucosal-activated invariant T cells do not exhibit significant lung recruitment and proliferation profiles in macaques in response to infection with *Mycobacterium tuberculosis* CDC1551. Tuberculosis.

[30] Kauffman, K. D. et al. Limited pulmonary mucosal-associated invariant T cell accumulation and activation during *Mycobacterium tuberculosis* infection in Rhesus Macaques. *Infect. Immun.* **86**, e00431–18 (2018). 10.1128/IAI.00431-18 PMC6246904 30201702

[31] Cruz A (2010). Pathological role of interleukin 17 in mice subjected to repeated BCG vaccination after infection with *Mycobacterium tuberculosis*. J. Exp. Med.

[32] Bold TD, Banaei N, Wolf AJ, Ernst JD (2011). Suboptimal activation of antigen-specific CD4+ effector cells enables persistence of M. tuberculosis in vivo. PLoS Pathog..

[33] Moguche AO (2017). Antigen availability shapes T cell differentiation and function during tuberculosis. Cell Host Microbe.

[34] Urdahl KB, Shafiani S, Ernst JD (2011). Initiation and regulation of T-cell responses in tuberculosis. Mucosal Immunol..

[35] Srivastava S, Grace PS, Ernst JD (2016). Antigen export reduces antigen presentation and limits T cell control of *M. tuberculosis*. Cell Host Microbe.

[36] Srivastava S, Ernst JD (2014). Cell-to-cell transfer of *M. tuberculosis* antigens optimizes CD4 T cell priming. Cell Host Microbe.

[37] Griffiths KL (2016). Targeting dendritic cells to accelerate T-cell activation overcomes a bottleneck in tuberculosis vaccine efficacy. Nat. Commun..

[38] Meierovics AI, Cowley SC (2016). MAIT cells promote inflammatory monocyte differentiation into dendritic cells during pulmonary intracellular infection. J. Exp. Med..

[39] Croxford JL, Miyake S, Huang YY, Shimamura M, Yamamura T (2006). Invariant V(alpha)19i T cells regulate autoimmune inflammation. Nat. Immunol..

[40] Schaefer K, Albers J, Sindhuwinata N, Peters T, Meyer B (2012). A new concept for glycosyltransferase inhibitors: nonionic mimics of the nucleotide donor of the human blood group B galactosyltransferase. Chembiochem.

[41] Mak JY (2017). Stabilizing short-lived Schiff base derivatives of 5-aminouracils that activate mucosal-associated invariant T cells. Nat. Commun..

[42] Li K (2018). Synthesis, stabilization, and characterization of the MR1 ligand precursor 5-amino-6-D-ribitylaminouracil (5-A-RU). PLoS One.

[43] Rao GV (2003). Efficacy of a technique for exposing the mouse lung to particles aspirated from the pharynx. J. Toxicol. Environ. Health A.

