# Supplementary material for: MAIT cell-directed therapy of Mycobacterium tuberculosis infection
Source: Mucosal Immunol. 2020 Aug 18;14(1):199–208. doi: 10.1038/s41385-020-0332-4 (PMC7790750; doi:10.1038/s41385-020-0332-4)
Supplement: Supplementary file 1 — Supplementary Figures [file 41385_2020_332_MOESM1_ESM.pdf]

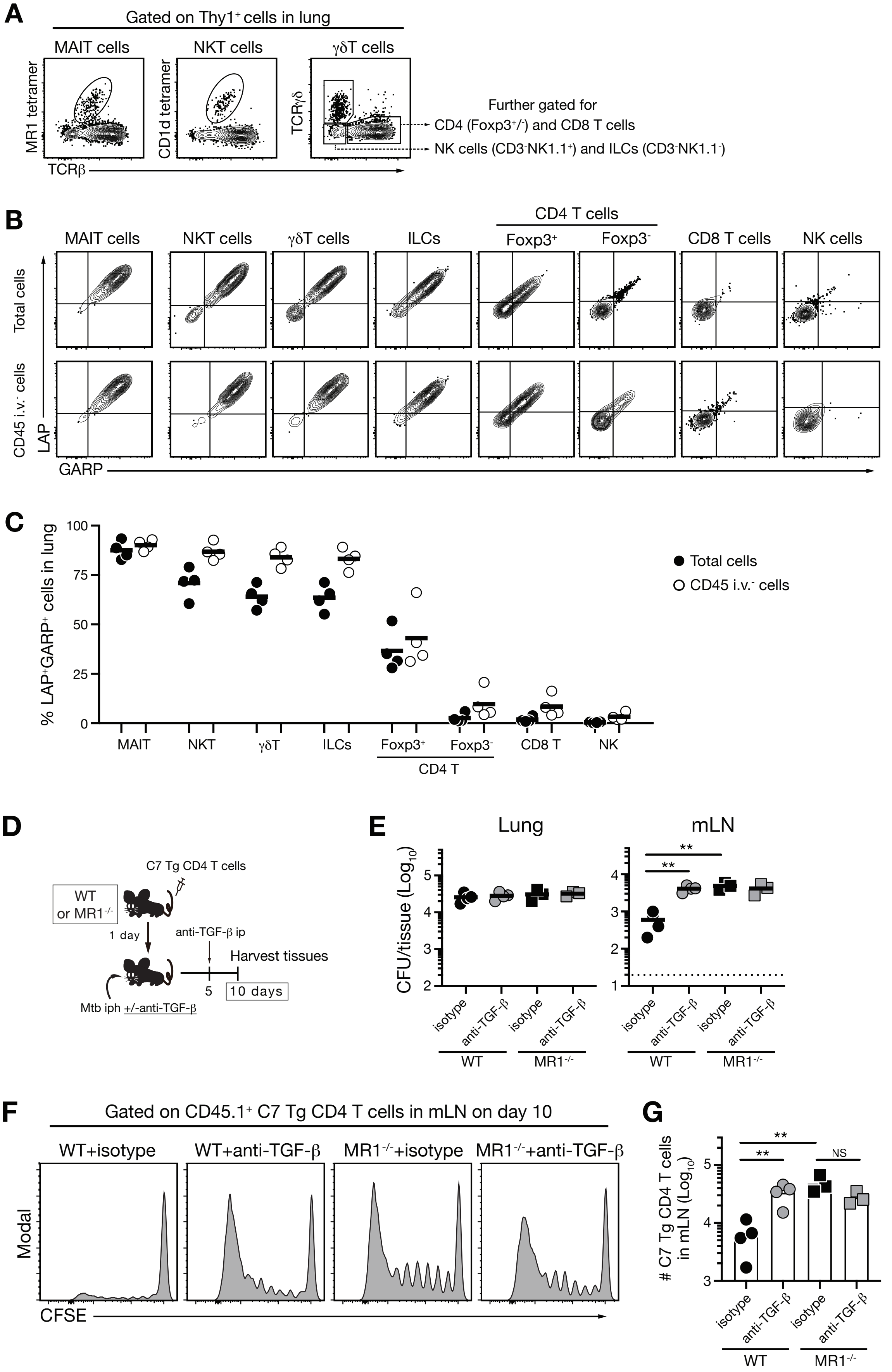

Figure S1. TGF-β blockade does not further accelerate CD4 T cell priming in MR1$^{-/-}$ mice.
(A) Gating strategy for LAP and GARP expression on lung lymphocytes in naive mouse. **(B-C)** Representative FACS plots (B) and frequency of LAP$^+$GARP$^+$ total (*top*) and CD45 i.v.$^-$ (*bottom*) cells in naive lungs (C). **(D)** CFSE-labeled C7 T CD4 T cells were adoptively transferred into WT or MR1$^{-/-}$ mice 1 day before iph Mtb infection. Mice were treated with isotype or anti-TGF-β antibody at the time of infection and 5 days p.i., then tissues were harvested on day 10 p.i. **(E-G)** Bacterial CFU in the tissues (E), representative histograms of CFSE dilution (F) and number (G) of C7 CD4 T cells in the mLN from mice treated as shown in (D). $^{**}p < 0.01$; NS, not significant.

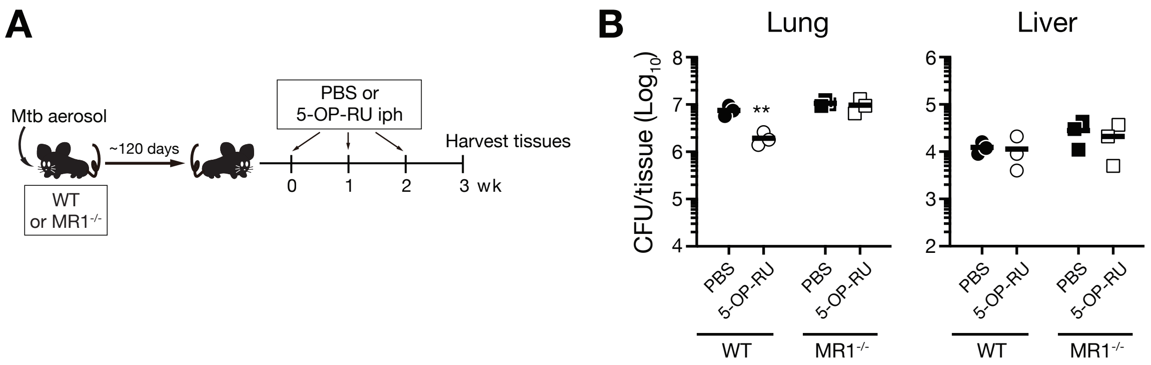

Figure S2. Therapeutic effect of 5-OP-RU in chronic Mtb infection is MR1 depedent.
(A) Mtb-infected WT or $MR1^{-/-}$ mice (~120 days p.i.) were iph treated with PBS or 5-OP-RU once a week for 3 weeks. Tissues were harvested a week after the last treatment. **(B)** Bacterial CFU in the tissues. $^{**}p < 0.01$.
